# Wastewater-Based Epidemiology for Infectious Diseases: A New Trick for an Old Threat

**DOI:** 10.1021/envhealth.6c00017

**Published:** 2026-06-04

**Authors:** Aurora Hirvonen, Laura Pellegrinelli, Sandro Binda, Elena Pariani

**Affiliations:** † Department of Public Health, Experimental and Forensic Medicine, University of Pavia, 27100 Pavia, Italy; ‡ Department of Biomedical Sciences for Health, 9304 University of Milan, 20122 Milano, Italy

**Keywords:** wastewater-based epidemiology, public health, wastewater surveillance, infectious disease, epidemiological modeling

## Abstract

Wastewater-based epidemiology (WBE) is an innovative approach to epidemiology that offers unique opportunities for public health surveillance. Its potential had been recognized in various applications over the years, but it was the global scale of the response to the SARS-CoV-2 pandemic that truly brought WBE to the fore. In this perspective paper we explore the untapped potential of WBE as a catalyst for infectious disease surveillance and as a One Health epidemiological tool, and the future horizons and innovative applications of WBE. It is clear that WBE will address a growing number of pathogens of concern to human health, such as avian influenza viruses, mpox, enterovirus D68, *Candida auris*, and antimicrobial resistance. In addition, it will contribute to epidemic intelligence by monitoring mass gathering events, and by predictive modeling and forecasting in combination with artificial intelligence to mitigate and prevent infectious diseases from reaching the highest level of clinical complexity. We believe that the maximum performance and complete institutional integration into public health of WBE is yet to be realized on a global scale.

## Graphical abstract

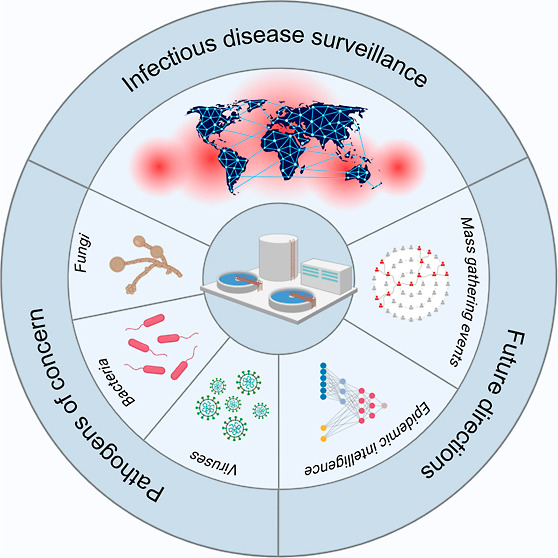

## Introduction

Wastewater-based epidemiology (WBE) is a relatively novel approach. Although wastewater-based surveillance has had a number of applications over the years, its expansion has remained relatively niche. The COVID-19 pandemic, caused by the novel coronavirus, SARS-CoV-2, brought WBE to an unparalleled scale, as shown by the surge in WBE activity worldwide. Time and again, literature has confirmed how WBE reaches its ultimate potential when used as an epidemiological and public health tool for infectious disease surveillance and control.^1^ However, it is yet to be employed to its full potential in public health on a global scale. On this preface, we would like to present the SARS-CoV-2 pandemic as a field experiment that provided a solid foundation of observations, validations, and lessons learned. Now, with the knowledge acquired over the past five years (2020–2025), it is time to turn our gaze from the past to the future. It is time to move beyond what has already been stated and anchor WBE in an approach with the ability to forecast events of concern for public health, and mitigate and prevent infectious cases from reaching the highest level of clinical complexity. If implemented correctly, wastewater data could become the leading indicator of traditional surveillance data time series and a front-line tool for providing early warning of clinical threats.

**Table 1:** Strengths and Limitations of Wastewater-Based Epidemiology

strengths	limitations
representative of population-level data	low concentration and viability of pathogens in wastewater samples
near-real-time insights	detection of multiple genotypes hindered by first-generation sequencing methods
cost-effective and financially sustainable over time	analytical challenges due to the complexity of the wastewater matrix
representative of asymptomatic, presymptomatic and symptomatic individuals	confounded by variability in virus-specific and/or host-specific factors
early detection of recognized, re-emerging and novel pathogens	impact of the environment on pathogen detection and viability
flexible and adaptable surveillance approach	population size is difficult to estimate
maintains the privacy and anonymity of individuals within a population	unsewered populations are not directly addressed
guarantees social distancing during periods of high contagiousness	comparisons hindered by unstandardized sampling approaches, analysis methods and reporting standards

### Beyond and Above: WBE, a Valuable Public Health Tool

The true value of WBE is realized when it is adopted as a public health tool for infectious disease control. This can be achieved by aggregating its various applications, such as population-wide and disease-specific surveillance, the early detection of outbreaks and the tracking of variants of concern.^1^ These elements inform us about variations in disease frequency and pathogen composition across populations, space and time, and guide adequate public health responses, policy decisions and resource allocation.

It should be stressed how the value of WBE is not limited to retrospective measurements of disease prevalence, occurrence, spread or resurgence; it can also be used to forecast the evolution of pathogens before events of concern occur. Indeed, this can be the determining factor between a contained outbreak and an unmanageable epidemic or pandemic. WBE can challenge the long-held belief that endemic, seasonal, emergent or re-emergent infectious diseases are unpredictable.

Clinical cases remain the gold standard for the detection of many pathogens. Unfortunately, clinical data is hindered by reporting and behavioral biases around testing, as well as an inability to survey asymptomatic carriers.^2,3^ It is well established that the circulation of pathogens in sewage is directly comparable to disease prevalence in the population.^4,5^ Contrarily to clinical testing, where one sample provides information about a single individual, one wastewater sample provides spatial and temporal information on the entire contributing population. Therefore, WBE complements case-based surveillance, providing a true epidemiological picture. Figure 1 shows the placement of WBE within the wider public health surveillance pyramid.

**Figure 1:**
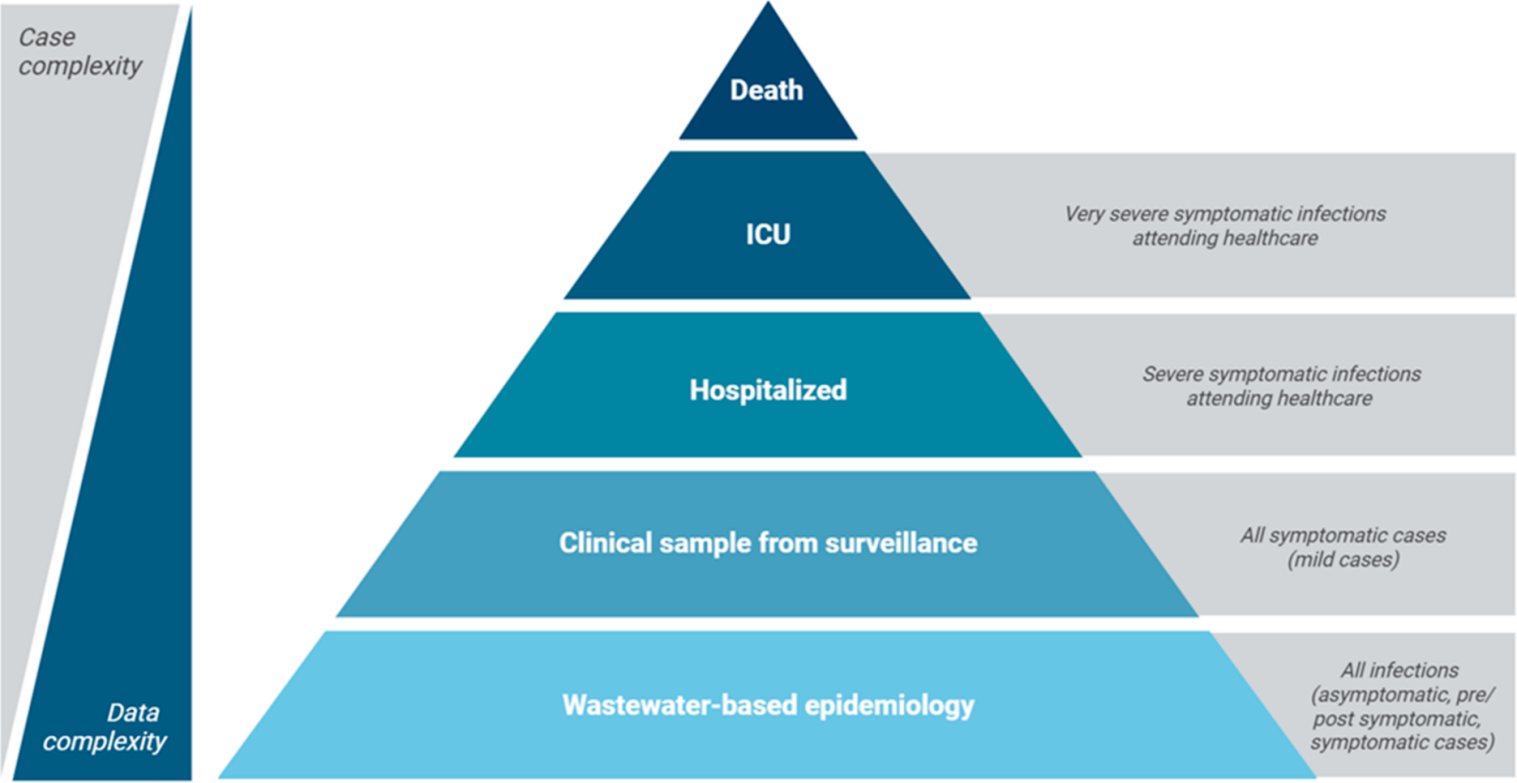
Public health surveillance pyramid and its relationship with clinical case and public health data complexity. Adapted from “Environmental surveillance for SARS-CoV-2 to complement other public health surveillance”, World Health Organization (2022). ICU = intensive care unit.

Existing clinical surveillance repeatedly underestimates, asymptomatic individuals in the population. However, these individuals play a significant role in disease transmission.^6^ It is important to consider pathogens that cause mild to no symptoms, but severe outcomes in a subset of the population. One such pathogen is enterovirus D68 (EV-D68), a polio-like virus known to cause acute flaccid myelitis (AFM) in children.^7,8^ Moreover, as demonstrated by the SARS-CoV-2 pandemic, periods of self-testing result in reduced availability of clinical case data. In both cases wastewater surveillance continued to provide key information, and fill important data gaps.^7,9^ The identification of viral transmission before symptom onset demonstrates how WBE foreshadows corresponding clinical data. As lead times can anticipate clinical events by up to a month, curbing endemics becomes possible.^10,11^

For the time being, WBE cannot be used as a stand-alone public health surveillance tool.^12^ On its own, it provides only partial information and requires other epidemiological nonwastewater indicators to identify correlations. These indicators extend beyond clinical data and include hospitalization counts, medical records, prescription or sales data, and morbidity and mortality rates^1,13^ Considering the broader context of surveillance data is critical so as to maximize the value of WBE and ensure accurate and specific data outputs. Moving forward, it can be said that the isolated use of WBE is not the objective of the evolving approach. Rather, investments should be directed toward enhancing the predictive capabilities of WBE to strengthen the integration and interpretation of data alongside different parameters.

### Strengths and Limitations of WBE for Infectious Diseases

WBE encompasses most of the characteristics that a surveillance system of the present and the future should have. As previously mentioned, WBE can detect and monitor the presence and changing quantities of a pathogen in populations which are not captured through routine clinical surveillance.^6^ It provides a dynamic snapshot of spatial and temporal patterns, such as the timing of the onset of shedding, the period of infectiousness and the clinical course. All this data is unattainable through single individual clinical testing.^11^ In fact, WBE represents the full spectrum of disease, including asymptomatic, presymptomatic and symptomatic cases.^7^ Furthermore, the prevalence data extracted using WBE precede corresponding clinical detections.^5^ This implies early detection of recognized, re-emerging and novel pathogens from 1 week to 1 month in advance.^10^

Furthermore, if data is required immediately, as in the case of an ongoing epidemic or pandemic, WBE provides near real-time insights. Trends can be reported more quickly: within 24 h of sample collection and as little as five to 7 days after toilet flushing. Trends can be disseminated more frequently and more consistently than with conventional surveillance.^7,14^ The real-time framework of WBE enables it to respond to current public health needs in terms of sampling location and frequency. Furthermore, its flexibility permits to move quickly when the need arises and to scale up for wide-scale population coverage. Besides, wastewater sampling always allows social distancing, even during periods of high contagiousness.

WBE is often more cost-effective than active individual testing-based surveillance, especially in larger and more heterogeneous populations, since one sample represents a pool of the population.^15^ In fact, a recent modeling study carried out in Germany found that wastewater-based surveillance can achieve a level of accuracy similar to that of clinical testing at about one-10th of the cost.^16^ In addition, the costs of WBE have been shown to be more stable over time and are expected to decrease as programmes continue to evolve and expand.^17^ Although the costs of WBE vary considerably between and within countries, they can be easily managed based on the choice of sample testing method, the transportation approach, the sampling frequency, and the size of the population covered, making the surveillance approach accessible to regions with fewer resources and disadvantaged communities.^16,17^ In this context, wastewater monitoring could help to reduce the data gap between low- and middle-income countries (LMIC) and high-income countries (HIC).

Another advantage of this approach is that WBE has the potential to expand the population represented. WBE is particularly pertinent to unique, at-risk, or vulnerable communities that are likely to be missed by conventional surveillance approaches.^5,7^ It overlooks population demographics such as gender, ethnicity, economic status and educational background and, hence, issues like privacy, anonymity of individuals, and stigmatization within some subpopulations are overcome.^1,13^ However, this makes it difficult to estimate the population covered, as it captures anyone contributing to the sewers, including transient populations, which do not remain constant over time.^14^ Furthermore, wastewater surveillance cannot provide information on unsewered populations, posing logistical challenges for sample collection, especially in LMICs with limited or absent sewer infrastructure. In populations with sufficient data from served communities, trends can be extrapolated; otherwise more targeted, decentralized, community-based approaches need to be adopted.^18,19^

As there is no perfect approach to surveillance, also WBE has some drawbacks and challenges. The low concentration and viability of pathogens in wastewater is a well-known limitation of WBE.^18^ The limit of detection for wastewater surveillance is yet to be agreed upon. In addition, the complexity of pathogen genomes and the unclassified status of many sequences remain challenges.^1^ Furthermore, the presence of multiple genotypes in the same sample hinders detection especially by Sanger sequencing and more advanced methods, such as next-generation sequencing need to be adopted instead.^10,13^ Unfortunately, these technologies may not be accessible to all, especially to laboratories residing in LMIC.

The complex nature of the wastewater matrix can also present analytical challenges.^1^ Unstandardized sampling approaches, non-normalized analysis methods and varied reporting standards can produce contrasting epidemiological trends that are difficult to compare.^7^ Furthermore, the interpretation of data may be complicated by variability in virus-specific factors, such as shedding levels and pathogen stability, as well as by host-specific factors including age, symptoms, and immunity.^1,6^ While the evidence base for fecal shedding of enteric viruses is well established, studies on nonenteric viruses remain limited. Fecal shedding dynamics may not necessarily align with those at other sites, as shedding may begin in the presymptomatic phase and continue after the infectious period, when clinical samples result negative.^20^ Furthermore, there is scant research on how shedding varies according to age of the infected individual, comorbidities, vaccination status or reinfection. A better understanding of the impact of immunity (from vaccination or previous infection) could potentially improve the interpretation of epidemiological estimates throughout different phases of an outbreak.

Finally, as environmental samples, wastewater samples are subject to environmental influences. The persistence of biomarkers in wastewater is determined by physiochemical characteristics of the pathogens, temperature, seasonality and the presence of degrading components.^6,21^ For example, nonenveloped viruses tend to be more stable in the environment than enveloped viruses, which can survive for months in the environment.^6^ Furthermore, most current WBE studies do not consider temperature or seasonality, which can lead to misquantification of results and limit the comparability of data at different times of the year and in different regions of the world.^22^ Strengths and limitations of WBE are summarized in Table 1.

### The Implementation of WBE Against Other Concerning Pathogens

#### Avian Influenza Virus

The detection of influenza viruses in wastewater has become increasingly documented globally, particularly for type A virus due to its greater public health impact, role in the seasonal epidemics, and capacity to spillover from animal reservoirs.^23^ In 2024, the US experienced an outbreak of the highly pathogenic avian influenza A virus (IAV) H5N1 in livestock, with 67 human cases reported.^24^ The H5 hemagglutinin gene was detected through molecular methods in wastewater in multiple US states, including Texas, North Carolina, California, and Oregon, which overlapped with the ongoing IAV H5N1 outbreak.^25–27^ As a result, in May 2024, the Centers for Disease Control and Prevention (CDC) initiated the first national IAV wastewater monitoring program.^28,29^ While monitoring of H5 IAV gene in wastewater has proven to be an important surveillance indicator, current wastewater detection methods fail to discriminate between different IAV subtypes and strains, potentially capturing low-pathogenic H5 IAV as well. Furthermore, these methods cannot determine the species or the sources spreading H5 IAV, which could be human, animal, or animal-derived, such as milk from an infected cow.^28,30^ Indeed, various studies have concluded that the presence of H5 in wastewater is probably more related to virus-infected herds than to the excretion from infected humans.^26,28,31^ Combining detection assays with host-associated fecal markers, implementing metagenomic or targeted sequencing, and integrating wastewater results with veterinary and clinical surveillance are just some of the actions that public health officials can undertake to improve the differentiation of H5 IAV sources in wastewater.^32–34^ Furthermore, a deeper understanding of the contribution of human, animal and agricultural to the sewer system is required to correctly identify and interpret threats at the human–animal interface.

#### Monkeypox

When in February 2022 routine wastewater surveillance identified poliovirus in the wastewaters of London, many were startled by the fact that the virus, which was eradicated in Europe in 2003, had re-emerged in the region.^35,36^ Concurrently, in mid-2022 the detection of monkeypox virus (MPXV) in wastewater samples in the USA and Europe confirmed the potential of WBE to alert us on old and new threats.^37–41^ The recent cryptic detection of MPXV beyond its endemic boundaries led the World Health Organization to declare the outbreak a public health emergency of international concern.^42^ Monkeypox outbreaks have predominantly affected gay, bisexual, transgender and other gender-diverse people who have sex with men.^43^ Unlike WBE, clinical surveillance has been limited by the social stigma, discrimination, and racism surrounding mpox, which in turn underestimates the true prevalence and burden of the disease.^44^ Wastewater surveillance can overcome issues such as unwillingness to get tested, personal consent, underrepresentation of asymptomatic and mild clinical cases, and costs of testing. One anonymous pooled sample informs on community-level circulation data without disclosing individual identities.^40,44^ WBE has shown promising results in HIC for the early detection and monitoring of this nonenteric, nonrespiratory virus. Building on these results, the WBE approach should now be extended to regions where the virus is endemic, with the ultimate goal of achieving global eradication of monkeypox.

#### Enterovirus D68

WBE has become one of the most popular and fastest growing data sources for identifying enterovirus circulation in the population. Initially applied in the context of the Global Polio Eradication Initiative, its use has since been extended to other nonpolio enteroviruses spread by the fecal-oral route.^45,46^ Over the past decade, EV-D68 has emerged as a high-concern pathogen for public health due to its association with unprecedented cases of a polio-like paralysis called AFM in young children.^7,8^ Its distinct biennial circulation pattern and atypical enteroviral characteristics make the virus a possible candidate for surveillance. Although EV-D68 has been rarely found in stool samples from clinical cases, its detection in wastewater has been shown to be analytically feasible and successful.^47–55^ While there are still limited publications on its application, all available studies highlight the value of wastewater surveillance for EV-D68 during periods of clinical prevalence.^47–55^ Given the insufficient public health data on this pathogen, WBE can serve as an early warning signal and provide insight into the evolving epidemiological patterns of EV-D68, as well as evidence of silent subclinical circulation of the virus in the absence of paralytic disease.

#### Candida auris

As WBE has gained more attraction following the SARS-CoV-2 pandemic, the possibility of expanding the technology to nonviral pathogens has been evaluated more closely. One interesting pathogen that has been investigated is *Candida auris* (*C. auris*), an emerging opportunistic fungal pathogen (yeast) that is known to cause infections ranging from mild and superficial to severe and invasive.^7,56^ *C. auris* primarily colonizes long-term healthcare settings and nursing homes, which accommodate individuals who are most susceptible to severe complications from the infection. In 2020, a research has demonstrated that Candida species could be detected in hospital wastewater.^57^ However, it was only in 2023 that the connection between *C. auris* and community wastewater was further explored.^56^ The first study by Barber at al. (2023) and subsequent studies have provided proof of concept that *C. auris* can be detected in wastewater using quantitative PCR, and that there is a positive correlation between wastewater detections and wastewater treatment plant serving healthcare facilities involved in outbreaks.^56,58–60^ Prior to these findings, *C. auris* surveillance seemed to have reached a dead end. Conventional surveillance methods and the lack of alternative approaches have led to underestimate the true prevalence of infections. In this context, wastewater surveillance could help to improve our understanding of *C. auris* and assist in containing hospital- and community-scale outbreaks. Due to its multidrug resistance, persistence in clinical settings, and the high morbidity and mortality rates among infected individuals, *C. auris* is a significant public health concern. For now, the available data represents the early stage of implementation; however, by building upon these efforts and leveraging pre-existing wastewater surveillance frameworks, the narrative surrounding *C. auris* can be reshaped.

#### Antimicrobial Resistance

Antimicrobial resistance (AMR) is one of the most pressing global public health concerns of this decade. Drug-resistant pathogens reduce the effectiveness of antimicrobial treatments, leading to increased morbidity and mortality, prolonged hospital stays, and exponential healthcare costs.^61^ Increased international mobility has also contributed to the emergence of previously unidentified antimicrobial resistant pathogens in a number of countries.^62,63^ In a One Health context, the application of wastewater surveillance to monitor AMR dynamics at the human community level, among animals and in the environment has been promising.^64,65^ Yet, surveillance has been limited and exploratory in nature, focusing primarily on antibiotic-resistant bacterial pathogens. Furthermore, the translation and integration of these results into clinical surveillance and public health response systems has been slow, as AMR surveillance activities have primarily been conducted as short-term research projects with limited financial support.^65^ As new drug-resistant threats continue to emerge, wastewater can provide information on the drivers of AMR, identify hotspots for the evolution and spread of clinically relevant AMR determinants, provide insight on the effectiveness of currently used antimicrobials, guide the development of new treatment regimens, and inform data-driven policy action.^65^

#### A Stress Test: the Contribution of WBE in Controlling Mass Gathering Events

A practical application of WBE which is still under-considered is its use in controlling mass gathering (MG) events. Regardless of the nature or size of the event, the unusually dense concentration of people in one place at one time introduces the risk of infectious disease transmission. In addition, large international MG events increase global travel.^66^ The considerable influx and outflux of international travelers increases the possibility of disease importation from host countries, as well as the possibility of unexpected cross-border health issues.^67^ In these scenarios, the speed of detection and reporting–in as little as 24 h–are major strengths of WBE and are crucial for swift decision-making regarding the introduction of appropriate countermeasures. Hence, WBE can efficiently, rapidly and economically facilitate tracking of potential transmission chains of pathogens and their variants.

In recent years, wastewater surveillance has been successfully applied to the FIFA World Cup Qatar 2022 (FWC’22) and the Paris 2024 Summer Olympic and Paralympic Games (OPG).^66,67^ Qatar, a small country with a population of 2.7 million hosted over 1.7 million attendees amid the SARS-CoV-2 pandemic, while the 2024 Paris OPG saw an estimated 11.2 million spectators and 10,500 athletes, making it the largest sporting event held in the country to date.^67^ Both countries invested considerable time and effort in preparing, planning and evaluating priority pathogen targets for survey in wastewater. This plan was largely informed by the Tokyo 2020 OPG, which took place from July to September 2021 amid the ongoing global pandemic. During this period, Japan experienced an unexpected surge in cases of the novel SARS-CoV-2 Delta variant.^68^ The Tokyo OPG highlighted the strain and chaos that arise when an infectious agent is circulating uncontrollably within the population. This demonstrated the inadequacy of clinical screening during MG events, and the consequences of handoff unmanageable infection surveillance for the local population, including an inability to access testing and medical support.^68^ Thus, it can be said that MG events are a true stress test for the public health system, the allocation of resources and the local community’s timely scientific-based decision-making. The challenge lies in ensuring the health and safety of the millions of national and international attendees, including spectators, athletes, organizers, and the media. The wastewater surveillance plans drafted for the FWC’22 and the Paris 2024 OPG provide a foundation for future MG events, including large religious gatherings, to adopt WBE.

### Looking Forward: the Future Direction of WBE

Wastewater surveillance has produced robust and reliable results in the retrospective investigation of disease outbreaks. The conclusion is always the same: WBE reaches its ultimate potential when used as an epidemiological and public health tool for infectious disease surveillance and control. With extensive proof of concept, investments should now be made to increase the strong predictive and analytical capabilities of WBE, thereby limiting infectious cases from reaching their highest levels of clinical complexity. Deep learning, artificial intelligence (AI), machine learning (ML), predictive algorithms and statistical models are just some of the automated, data-driven systems that are paving the way for the future. Preliminary research has demonstrated the success of wastewater modeling in the nowcasting and forecasting of epidemiological trends.^69–72^

In a recent systematic review, Wang et al. (2026) examined the current state of WBE modeling.^73^ The authors highlight that the complexity of these models stems from selecting the most pertinent subset of features for analysis. Wastewater surveillance data combined with clinical data, including case counts and hospitalization, form the backbone of all WBE modeling strategies. However, a wide range of additional covariates can further enhance model performance. These may include epidemiological, behavioral, policy-related, wastewater-derived, socioeconomic, environmental, and temporal indicators, depending on the specific application of the model.^73^ Data availability and granularity remain challenges for their robust and practical implementation.

To date, mathematical models based on wastewater have been almost exclusively explored for SARS-CoV-2, with very few advances in relation to other infectious diseases.^69,70,74^ In this light, Wang et al. (2026) have proposed a scenario-based decision-making framework for model selection in WBE.^73^ The framework outlines seven distinct public health surveillance scenarios, ranging from early outbreak detection to routine monitoring and from high-to low-resource settings. It is intended to serve as a high-level guidance tool for robust, actionable and context-aware modeling strategies.^73^ Importantly, the framework expands the potential application of wastewater-based modeling to a broader range of pathogens detectable in wastewater–beyond SARS-CoV-2–and helps contextualize the selection of suitable covariates by referencing existing examples.^73^

Ultimately, data-driven-surveillance systems may provide standardized metrics, including population-adjusted pathogen loads, indicators of directional and magnitude change, estimated infection prevalence, and data quality measures. The metrics can support workflows and best practices that can be easily reproduced anywhere in the world. At the same time, they operationalize more equitable forms of knowledge transfer.^5,^ ^75^ In this context, AI and ML models have the potential to support multiscale monitoring and transform data into effective outputs that can provide innovative solutions to current and future threats. These concepts could change the long-held belief that infectious diseases are dynamic and thus not fully predictable. WBE is set to become the front-line indicator of traditional surveillance data time series. However, it is up to us to take the lead in anticipation of the next big thing. Such efforts could determine whether a disease outbreak remains contained or escalates into an unmanageable epidemic or even a pandemic.

## Abbreviations

AFM: acute flaccid myelitis
AI: artificial intelligence
AMR: antimicrobial resistance
*C. auris*: *Candida auris*
CDC: Centers for Disease Control and Prevention
EV-D68: enterovirus D68
FWC’22: FIFA World Cup Qatar 2022
HIC: high-income countries
IAV: avian influenza A virus
LMIC: low- and middle-income countries
MG: mass gathering
ML: machine learning
MPXV: monkeypox virus
OPG: Olympic and Paralympic Games
WBE: wastewater-based epidemiology

## References

[1] O’Keeffe J. (2021). Wastewater-Based
Epidemiology: Current Uses and Future Opportunities as a Public Health
Surveillance Tool. Environ. Health Rev..

[2] Castiglioni S., Schiarea S., Pellegrinelli L., Primache V., Galli C., Bubba (2022). SARS-CoV-2
RNA in Urban Wastewater Samples to Monitor the COVID-19 Pandemic in
Lombardy, Italy (March–June 2020). Sci.
Total Environ..

[3] Ritchey M. D., Rosenblum H. G., Del Guercio K., Humbard M., Santos S., Hall J. (2022). COVID-19 Self-Test Data: Challenges and Opportunities
- United States, October 31, 2021-June 11, 2022. MMWR Morb Mortal Wkly Rep.

[4] Delogu R., Battistone A., Buttinelli G., Fiore S., Fontana S., Amato C. (2018). Poliovirus and Other Enteroviruses from Environmental
Surveillance in Italy, 2009–2015. Food
Environ. Virol..

[5] Levy J. I., Andersen K. G., Knight R., Karthikeyan S. (2023). Wastewater
Surveillance for Public Health. Science.

[6] Robins K., Leonard A. F. C., Farkas K., Graham D. W., Jones D. L., Kasprzyk-Hordern B. (2022). Research Needs for Optimising Wastewater-Based
Epidemiology Monitoring for Public Health Protection. J. Water Health.

[7] National Academies of Sciences, Engineering, and Medicine Wastewater-Based Disease Surveillance for Public Health Action; National Academies Press: Washington, D.C., 2023; . 10.17226/26767. 37184191

[8] Holm-Hansen C. C., Midgley S. E., Fischer T. K. (2016). Global Emergence of Enterovirus D68:
A Systematic Review. Lancet Infect. Dis..

[9] Zhang Y., Cen M., Hu M., Du L., Hu W., Kim J. J. (2021). Prevalence and Persistent
Shedding of Fecal SARS-CoV-2 RNA in Patients
With COVID-19 Infection: A Systematic Review and Meta-Analysis. Clin. Transl. Gastroenterol..

[10] Pellegrinelli L., Galli C., Seiti A., Primache V., Hirvonen A., Schiarea S. (2023). Wastewater-Based Epidemiology
Revealed in Advance
the Increase of Enterovirus Circulation during the Covid-19 Pandemic. Sci. Total Environ..

[11] Gagliano E., Biondi D., Roccaro P. (2023). Wastewater-Based
Epidemiology Approach:
The Learning Lessons from COVID-19 Pandemic and the Development of
Novel Guidelines for Future Pandemics. Chemosphere.

[12] The Potential of Wastewater-Based Epidemiology. Nat. Water 2023, 1 (5), 399. DOI: 10.1038/s44221-023-00093-6.

[13] Sims N., Kasprzyk-Hordern B. (2020). Future Perspectives of Wastewater-Based
Epidemiology:
Monitoring Infectious Disease Spread and Resistance to the Community
Level. Environ. Int..

[14] Boehm A. B., Hughes B., Duong D., Chan-Herur V., Buchman A., Wolfe M. K. (2023). Wastewater Concentrations
of Human Influenza, Metapneumovirus, Parainfluenza, Respiratory Syncytial
Virus, Rhinovirus, and Seasonal Coronavirus Nucleic-Acids during the
COVID-19 Pandemic: A Surveillance Study. Lancet
Microbe.

[15] Vitale D., Morales Suárez-Varela M., Picó Y. (2021). Wastewater-Based
Epidemiology, a Tool to Bridge Biomarkers of Exposure, Contaminants,
and Human Health. Curr. Opin. Environ. Sci.
Health..

[16] Wallrafen-Sam K., Zacharias N., Acero R. R., Walker A., Lukas M., Schneider B. (2025). EPH58 Evaluating the Cost-Effectiveness of
Wastewater-Based Disease Surveillance. Value
in Health.

[17] Hu, X. ; Keshaviah, A. ; Harrison, E. The Costs of Wastewater Monitoring in Low- and Middle-Income Countries; Mathematica: Washington, D.C., 2023.

[18] Kirby A. E., Walters M. S., Jennings W. C., Fugitt R., LaCross N., Mattioli M., Marsh Z. A., Roberts V. A., Mercante J. W., Yoder J. (2021). Using
Wastewater Surveillance Data to Support the COVID-19
Response — United States, 2020–2021. MMWR Morb Mortal Wkly Rep.

[19] Hamilton K. A., Wade M. J., Barnes K. G., Street R. A., Paterson S. (2024). Wastewater-Based
Epidemiology as a Public Health Resource in Low- and Middle-Income
Settings. Environ. Pollut..

[20] Zheng G., Chan E. M. G., Boehm A. B. (2025). Systematic Review and Meta-Analysis
of Enteric Virus Shedding in Human Excretions. EBioMedicine.

[21] Heijnen L., Medema G. (2011). Surveillance of Influenza
A and the Pandemic Influenza
A (H1N1) 2009 in Sewage and Surface Water in the Netherlands. J. Water Health.

[22] Hart O. E., Halden R. U. (2020). Simulated 2017 Nationwide Sampling at 13,940 Major
U.S. Sewage Treatment Plants to Assess Seasonal Population Bias in
Wastewater-Based Epidemiology. Sci. Total Environ..

[23] Viviani L., Vecchio R., Pariani E., Sandri L., Binda S., Ammoni E. (2025). Wastewater-Based
Epidemiology of Influenza Viruses:
A Systematic Review. Sci. Total Environ..

[24] CDC . Global Human Cases with Influenza A­(H5N1), 1997–2025. Avian Influenza (Bird Flu). https://www.cdc.gov/bird-flu/php/surveillance/chart-epi-curve-ah5n1.html (accessed Sep 25, 2025).

[25] Wolfe M. K., Duong D., Shelden B., Chan E. M. G., Chan-Herur V., Hilton S. (2024). Detection
of Hemagglutinin H5 Influenza A Virus Sequence
in Municipal Wastewater Solids at Wastewater Treatment Plants with
Increases in Influenza A in Spring, 2024. Environ.
Sci. Technol. Lett..

[26] Paulos A. P., Hilton S. P., Shelden B., Duong D., Boehm A. B., Wolfe M. K. (2025). Detection of Hemagglutinin H5 Influenza
A Virus RNA
and Model of Potential Inputs in an Urban California Sewershed. Environ. Sci. Technol..

[27] Falender R., Radniecki T. S., Kelly C., Cieslak P., Mickle D., Hall H. (2025). Avian Influenza A­(H5) Subtype in Wastewater - Oregon,
September 15, 2021-July 11, 2024. MMWR Morb
Mortal Wkly Rep.

[28] Honein M. A., Olsen S. J., Jernigan D. B., Daskalakis D. C. (2024). Challenges
and Opportunities for Wastewater Monitoring of Influenza Viruses During
the Multistate Outbreak of Highly Pathogenic Avian Influenza A­(H5N1)
Virus in Dairy Cattle and Poultry. Am. J. Public
Health.

[29] Louis S., Mark-Carew M., Biggerstaff M., Yoder J., Boehm A. B., Wolfe M. K. (2024). Wastewater Surveillance for Influenza A Virus
and H5 Subtype Concurrent with the Highly Pathogenic Avian Influenza
A­(H5N1) Virus Outbreak in Cattle and Poultry and Associated Human
Cases - United States, May 12-July 13, 2024. MMWR Morb Mortal Wkly Rep.

[30] Branda F., Ciccozzi M., Scarpa F. (2024). Tracking the Spread of Avian Influenza
A­(H5N1) with Alternative Surveillance Methods: The Example of Wastewater
Data. Lancet Infect. Dis..

[31] Tisza M. J., Hanson B. M., Clark J. R., Wang L., Payne K., Ross M. C. (2024). Sequencing-Based
Detection of Avian Influenza A­(H5N1)
Virus in Wastewater in Ten Cities. N. Engl.
J. Med..

[32] Hughes B., Beale D. J., Dennis P. G., Cook S., Ahmed W. (2017). Cross-Comparison
of Human Wastewater-Associated Molecular Markers in Relation to Fecal
Indicator Bacteria and Enteric Viruses in Recreational Beach Waters. Appl. Environ. Microbiol..

[33] Ren W., Rose J. B. (2025). Call for Expanding Environmental Surveillance of H5N1:
The Role of Microbial Source Tracking. Environ.
Sci. Technol. Lett..

[34] Louis S., Mark-Carew M., Biggerstaff M., Yoder J., Boehm A. B., Wolfe M. K., Flood M., Peters S., Stobierski M. G., Coyle J. (2024). Wastewater
Surveillance for Influenza A Virus and H5
Subtype Concurrent with the Highly Pathogenic Avian Influenza A­(H5N1)
Virus Outbreak in Cattle and Poultry and Associated Human Cases —
United States, May 12–July 13, 2024. MMWR Morb Mortal Wkly Rep.

[35] Wise J. (2022). Poliovirus
Is Detected in Sewage from North and East London. BMJ..

[36] Klapsa D., Wilton T., Zealand A., Bujaki E., Saxentoff E., Troman C. (2022). Sustained Detection of Type 2 Poliovirus in London
Sewage between February and July, 2022, by Enhanced Environmental
Surveillance. Lancet.

[37] Wolfe M. K., Yu A. T., Duong D., Rane M. S., Hughes B., Chan-Herur V. (2023). Use of Wastewater for Mpox Outbreak Surveillance
in California. N. Engl. J. Med..

[38] De
Jonge E. F., Peterse C. M., Koelewijn J. M., Van Der Drift A.-M. R., Van Der Beek R. F. H.
J., Nagelkerke E. (2022). The Detection of Monkeypox Virus DNA in Wastewater Samples in the
Netherlands. Sci. Total Environ..

[39] La
Rosa G., Mancini P., Veneri C., Ferraro G. B., Lucentini L., Iaconelli M., Suffredini E. (2023). Detection of Monkeypox Virus DNA
in Airport Wastewater, Rome, Italy. Emerging
Infect. Dis..

[40] Girón-Guzmán I., Díaz-Reolid A., Truchado P., Carcereny A., García-Pedemonte D., Hernáez B. (2023). Spanish Wastewater Reveals the Current Spread
of Monkeypox Virus. Water Res..

[41] Gazecka M., Sniezek J., Maciolek K., Kowala-Piaskowska A., Zmora P. (2023). Mpox Virus Detection in the Wastewater and the Number of Hospitalized
Patients in the Poznan Metropolitan Area, Poland. Int. J. Infect. Dis..

[42] WHO Director-General declares the ongoing monkeypox outbreak a Public Health Emergency of International Concern. https://www.who.int/europe/news/item/23-07-2022-who-director-general-declares-the-ongoing-monkeypox-outbreak-a-public-health-event-of-international-concern (accessed 11 13, 2024).

[43] Gaspari V., Rossini G., Robuffo S., Rapparini L., Scagliarini A., Mistral De Pascali A., Piraccini B. M., Lazzarotto T. (2023). Monkeypox Outbreak 2022: Clinical
and Virological Features
of 30 Patients at the Sexually Transmitted Diseases Centre of Sant’
Orsola Hospital, Bologna, Northeastern Italy. J. Clin. Microbiol..

[44] Tiwari A., Adhikari S., Kaya D., Islam M. A., Malla B., Sherchan S. P. (2023). Monkeypox Outbreak: Wastewater and Environmental
Surveillance Perspective. Sci. Total Environ..

[45] World Health Organization . Guidelines for Environmental Surveillance of Poliovirus Circulation; WHO/V&B/03.03; World Health Organization, 2003. https://apps.who.int/iris/handle/10665/67854 (accessed Mar 10, 2022).

[46] Centers for Disease Control and Prevention (CDC); World Health Organization. Regional Office for Europe . Enterovirus Surveillance Guidelines. Guidelines for Enterovirus Surveillance in Support of the Polio Eradication Initiative, 2015. https://www.euro.who.int/en/publications/abstracts/enterovirus-surveillance-guidelines.-guidelines-for-enterovirus-surveillance-in-support-of-the-polio-eradication-initiative (accessed Feb 17, 2022).

[47] Tedcastle A., Wilton T., Pegg E., Klapsa D., Bujaki E., Mate R. (2022). Detection of Enterovirus D68 in Wastewater Samples
from the UK between July and November 2021. Viruses.

[48] Weil M., Mandelboim M., Mendelson E., Manor Y., Shulman L., Ram D. (2017). Human Enterovirus D68 in Clinical and Sewage Samples
in Israel. J. Clin. Virol..

[49] Majumdar M., Wilton T., Hajarha Y., Klapsa D., Martin J. (2019). Detection
of Enterovirus D68 in Wastewater Samples from the United Kingdom during
Outbreaks Reported Globally between 2015 and 2018. bioRxiv.

[50] Erster O., Bar-Or I., Levy V., Shatzman-Steuerman R., Sofer D., Weiss L. (2022). Monitoring
of Enterovirus
D68 Outbreak in Israel by a Parallel Clinical and Wastewater Based
Surveillance. Viruses.

[51] Rector A., Bloemen M., Thijssen M., Pussig B., Beuselinck K., Van Ranst M. (2024). Respiratory Viruses in Wastewater Compared
with Clinical Samples, Leuven, Belgium. Emerg
Infect Dis.

[52] Boehm A. B., Wadford D. A., Hughes B., Duong D., Chen A., Padilla T. (2023). Trends
of Enterovirus D68 Concentrations in Wastewater,
California, USA, February 2021-April 2023. Emerg
Infect Dis.

[53] Boehm A. B., Wolfe M. K., Bidwell A. L., Zulli A., Chan-Herur V., White B. J. (2024). Human Pathogen Nucleic Acids in Wastewater
Solids from 191 Wastewater Treatment Plants in the United States. Sci. Data.

[54] Bisseux M., Colombet J., Mirand A., Roque-Afonso A.-M., Abravanel F., Izopet J. (2018). Monitoring
Human Enteric
Viruses in Wastewater and Relevance to Infections Encountered in the
Clinical Setting: A One-Year Experiment in Central France, 2014 to
2015. Eurosurveillance: European communicable
disease bulletin.

[55] Thongprachum A., Fujimoto T., Takanashi S., Saito H., Okitsu S., Shimizu H. (2018). Detection
of Nineteen Enteric Viruses in Raw Sewage
in Japan. Infect., Genet. Evol..

[56] Barber C., Crank K., Papp K., Innes G. K., Schmitz B. W., Chavez J. (2023). Community-Scale Wastewater
Surveillance of Candida
Auris during an Ongoing Outbreak in Southern Nevada. Environ. Sci. Technol..

[57] Mataraci-Kara E., Ataman M., Yilmaz G., Ozbek-Celik B. (2020). Evaluation
of Antifungal and Disinfectant-Resistant Candida Species Isolated
from Hospital Wastewater. Arch. Microbiol..

[58] Rossi A., Chavez J., Iverson T., Hergert J., Oakeson K., LaCross N. (2023). Candida
Auris Discovery through Community Wastewater
Surveillance during Healthcare Outbreak, Nevada, USA, 2022. Emerging Infect. Dis..

[59] Zulli A., Chan E. M. G., Shelden B., Duong D., Xu X.-R. S., White B. J. (2024). Prospective
Study of Candida Auris Nucleic
Acids in Wastewater Solids in 190 Wastewater Treatment Plants in the
United States Suggests Widespread Occurrence. mBio.

[60] Chavez J., Crank K., Barber C., Gerrity D., Iverson T., Mongillo J. (2024). Early
Introductions of Candida Auris Detected
by Wastewater Surveillance, Utah, USA, 2022–2023. Emerging Infect. Dis..

[61] Murray C. J. L., Ikuta K. S., Sharara F., Swetschinski L., Robles Aguilar G., Gray A., Han C., Bisignano C., Rao P., Wool E. (2022). Global
Burden of Bacterial Antimicrobial Resistance
in 2019: A Systematic Analysis. Lancet.

[62] Sridhar S., Turbett S. E., Harris J. B., LaRocque R. C. (2021). Antimicrobial-Resistant
Bacteria in International Travelers. Curr. Opin.
Infect. Dis..

[63] Tiwari A., Krolicka A., Tran T. T., Räisänen K., Ásmundsdóttir A. ´. M., Wikmark O.-G. (2024). Antibiotic
Resistance Monitoring in Wastewater in the Nordic Countries: A Systematic
Review. Environ. Res..

[64] Punch R., Azani R., Ellison C., Majury A., Hynds P. D., Payne S. J. (2025). The Surveillance of Antimicrobial Resistance
in Wastewater from a One Health Perspective: A Global Scoping and
Temporal Review (2014–2024). One Health.

[65] Pruden A., Vikesland P. J., Davis B. C., de Roda
Husman A. M. (2021). Seizing
the Moment: Now Is the Time for Integrated Global Surveillance of
Antimicrobial Resistance in Wastewater Environments. Curr. Opin. Microbiol..

[66] El-Malah S. S., Saththasivam J., K A. K., Abdul Jabbar K., Gomez T. A., Wahib S., Lawler J., Tang P., Mirza F., Al-Hail H. (2024). Leveraging Wastewater
Surveillance for Managing the Spread of SARS-CoV-2 and Concerned Pathogens
during FIFA World Cup Qatar 2022. Heliyon.

[67] Toro L., Valk H. de, Zanetti L., Huot C., Tarantola A., Fournet N. (2024). Pathogen
Prioritisation for Wastewater Surveillance
Ahead of the Paris 2024 Olympic and Paralympic Games, France. Eurosurveillance.

[68] Shimizu K., Mossialos E., Shibuya K. (2022). What Has the 2020 Tokyo Olympic and
Paralympic Games Taught Global Health on Sporting Mass Gatherings
under COVID-19 Pandemic?. Anaesth. Crit. Care
Pain Med..

[69] Fu S., Zhang Y., Li Y., Zhang Z., Du C., Wang R. (2024). Estimating
Epidemic Trajectories of SARS-CoV-2 and
Influenza A Virus Based on Wastewater Monitoring and a Novel Machine
Learning Algorithm. Sci. Total Environ..

[70] Ai Y., He F., Lancaster E., Lee J. (2022). Application of Machine Learning for
Multi-Community COVID-19 Outbreak Predictions with Wastewater Surveillance. PLoS One.

[71] Jeng H. A., Singh R., Diawara N., Curtis K., Gonzalez R., Welch N. (2023). Application of Wastewater-Based
Surveillance and Copula
Time-Series Model for COVID-19 Forecasts. Sci.
Total Environ..

[72] Nourbakhsh S., Fazil A., Li M., Mangat C. S., Peterson S. W., Daigle J. (2022). A Wastewater-Based
Epidemic Model for SARS-CoV-2 with
Application to Three Canadian Cities. Epidemics.

[73] Wang L., Amarasiri M., Oishi W., Sano D. (2026). From Wastewater
to
Epidemiological Insights: A Systematic Review of Modeling Strategies
for Infectious Disease Surveillance. Water Res..

[74] Hart O. E., Halden R. U. (2020). Computational Analysis of SARS-CoV-2/COVID-19
Surveillance
by Wastewater-Based Epidemiology Locally and Globally: Feasibility,
Economy, Opportunities and Challenges. Sci.
Total Environ..

[75] WHO . Preparedness and Resilience for Emerging Threats (PRET). World Heath Organization - WHO. https://www.who.int/initiatives/preparedness-and-resilience-for-emerging-threats (accessed Nov 01, 2023).

